# Ferromagnetic Interlayer Coupling in CrSBr Crystals Irradiated by Ions

**DOI:** 10.1021/acs.nanolett.3c01920

**Published:** 2023-09-05

**Authors:** Fangchao Long, Mahdi Ghorbani-Asl, Kseniia Mosina, Yi Li, Kaiman Lin, Fabian Ganss, René Hübner, Zdenek Sofer, Florian Dirnberger, Akashdeep Kamra, Arkady V. Krasheninnikov, Slawomir Prucnal, Manfred Helm, Shengqiang Zhou

**Affiliations:** † Helmholtz-Zentrum Dresden-Rossendorf, Institute of Ion Beam Physics and Materials Research, Bautzner Landstrasse 400, 01328 Dresden, Germany; ‡ TU Dresden, 01062 Dresden, Germany; § Department of Inorganic Chemistry, University of Chemistry and Technology Prague, Technická 5, 166 28 Prague 6, Czech Republic; ∥ University of Michigan-Shanghai Jiao Tong University Joint Institute, Shanghai Jiao Tong University, Shanghai, 200240, China; ⊥ Institute of Applied Physics and Würzburg-Dresden Cluster of Excellence ct.qmat, Technische Universität Dresden, 01069 Dresden, Germany; # Condensed Matter Physics Center (IFIMAC) and Departamento de Física Teórica de la Materia Condensada, Universidad Autónoma de Madrid, Ciudad Universitaria de Cantoblanco, 28049, Madrid, Spain

**Keywords:** 2D magnets, 2D semiconductor, Defects, Ion irradiation, Induced ferromagnetism

## Abstract

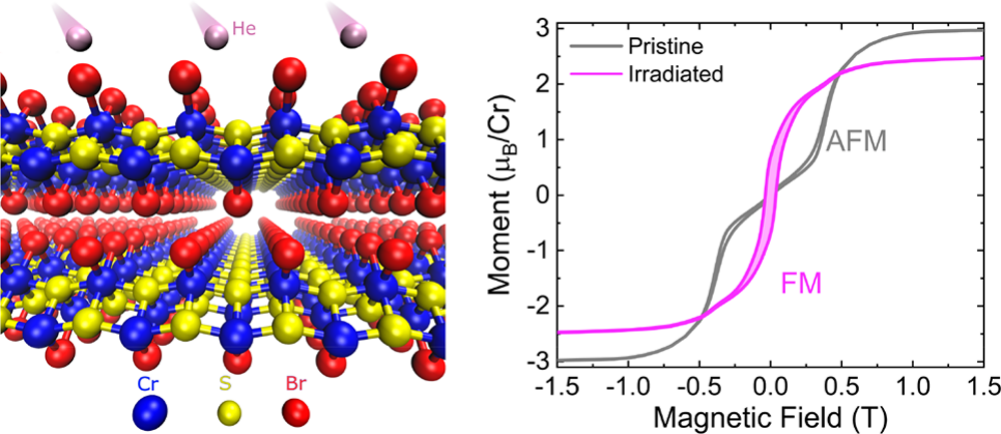

Layered magnetic materials are becoming a major platform for future spin-based applications. Particularly, the air-stable van der Waals compound CrSBr is attracting considerable interest due to its prominent magneto-transport and magneto-optical properties. In this work, we observe a transition from antiferromagnetic to ferromagnetic behavior in CrSBr crystals exposed to high-energy, non-magnetic ions. Already at moderate fluences, ion irradiation induces a remanent magnetization with hysteresis adapting to the easy-axis anisotropy of the pristine magnetic order up to a critical temperature of 110 K. Structure analysis of the irradiated crystals in conjunction with density functional theory calculations suggests that the displacement of constituent atoms due to collisions with ions and the formation of interstitials favors ferromagnetic order between the layers.

Van der Waals (vdW) magnets are attracting considerable interest from the scientific community. The ability to isolate single layers that can be reassembled into complex heterostructures makes them particularly useful for spin-based technologies.^1^ Two-dimensional (2D) ferromagnetism^2,3^ was first found in CrI_3_ and Cr_2_Ge_2_Te_6_, but these materials are not stable under ambient conditions.^4,5^ For instance, CrI_3_, a prototypical vdW magnet, deteriorates within minutes in air, which strongly hampers the fabrication and investigation of devices made from this compound. Hence, considerable efforts devoted to the search for new vdW magnetic crystals brought forth a number of air-stable antiferromagnetic (AFM) compounds, such as those from the *MPX*_3_ family (*M* = Mn, Fe, Ni; *X* = S, Se),^6^ and the layered magnetic semiconductor CrSBr.^7,8^ However, to exploit the full potential of vdW magnetic crystals for spin-based applications that require ferromagnetic (FM) coupling between magnetic moments, like magnetic memory devices, new experimental methods to control the magnetic coupling in vdW materials have to be developed.^9,10^

Due to the pronounced correlations between magnons, photons, electrons, and phonons,^7,11−14^ CrSBr is emerging as one of the most promising materials for such applications. Up to the Néel temperature of *T*_N_ = 132 ± 1 K, the FM spin order within each layer is compensated by an AFM arrangement of the vdW layers in the out-of-plane direction (*c*-axis),^15^ which is the reason for the vanishing net magnetization of pristine bulk crystals. First-principles calculations of magnetic moments localized on the Cr ions predict a magnetic order up to 160 K in a single layer.^7,16^ Second harmonic generation^17^ and magneto-transport measurements^7,18^ determined the FM order of a single CrSBr layer to be around 150 K. Overall, bulk CrSBr is characterized by a relatively weak AFM coupling between the vdW layers, which is also apparent from the fact that a small field of 0.4 T applied along the easy-axis (*b*-axis) is sufficient to switch the magnetic order from AFM to FM.^11^ The weak interaction between the layers makes CrSBr particularly susceptible to the modification of the magnetic order by external stimuli. Besides the recently demonstrated feasibility of strain and ligand substitution in altering the magnetic properties of CrSBr,^19−22^ ion irradiation may thus be a viable tool for modifying the magnetic structure with additional benefits, such as local patterning.

Here, we report a change of the magnetic interlayer coupling in bulk and few-layer CrSBr crystals irradiated with nonmagnetic ions. In the absence of an applied field and below a critical temperature of 110 K, the irradiated samples show sizable spontaneous magnetization which adapts to the easy-axis anisotropy of the pristine magnetic order and marks the transition of the magnetic ground state of irradiated CrSBr crystals from AFM to FM. Raman spectroscopy evidences the ion-fluence-dependent softening of the crystal lattice that is indicative of the formation of a large number of crystallographic defects. In conjunction with structure analysis, first-principles calculations suggest that the displacement of the atoms, particularly Cr, into interstitial positions between the vdW layers favors FM interlayer coupling. Our study highlights the potential of ion irradiation for nonchemical engineering of magnetism in vdW crystals.

Millimeter-sized bulk CrSBr crystals are synthesized using a chemical vapor transport method (see Supporting Information, Methods). Micro-Raman spectroscopy is performed using a linearly polarized, continuous-wave, 532 nm Nd:YAG laser for excitation, and magnetic properties are measured by a superconducting quantum interference device (Quantum Design, SQUID-VSM) magnetometer.

In our study, we subjected two types of CrSBr samples to He^+^ ion irradiation. The first type is a bulk crystal with a thickness of around 200 μm, the top surface of which is irradiated by 1.7-MeV He^+^ ions under different fluences. We start with an ion fluence of 4 × 10^14^/cm^2^ and monitor the ion-induced change in magnetic and structural properties using SQUID magnetometry and Raman spectroscopy. We then systematically repeat this experimental protocol, increasing the ion fluence at each step, until we reach a maximum fluence of 8 × 10^15^/cm^2^ (to indicate the fluence, the samples are named 4E14···8E15). To complement our investigation of this crystal, we study a second type of sample containing many small CrSBr flakes with a thickness of less than 1 μm that we obtain by exfoliation onto a silicon substrate. To achieve irradiation effects comparable to those in the large bulk crystal, we vary the energy of the He^+^ ions for the thin flake sample (see Supporting Information, Table S1).

As depicted in Figure 1a, each layer of CrSBr is composed of a buckled plane of CrS complexes surrounded by a sheet of Br atoms. Three characteristic modes, attributed to the A_g_ modes of the out-of-plane vibration, can be clearly identified in the Raman spectra (cf. Figure 1b,c,d).^23^ The A_g_^1^ (113.8 cm^–1^) and A_g_^3^ (342.5 cm^–1^) modes show maximum intensity when the laser polarization is parallel to the *b*-axis, while the A_g_^2^ (245.2 cm^–1^) mode is most pronounced when the polarization is aligned with the *a*-axis, reflecting the strong structural anisotropy of the crystal structure. Even CrSBr crystals exposed to the largest ion fluences applied in our study maintain their atomic structure and this anisotropy. The gradual decrease in the peak intensity of the Raman signatures and the continuous shift toward smaller wavenumbers observed in Figure 1c,d (also see Figure S1) are generally attributed to a softening of the phonon modes resulting from defect-induced variations in the lattice spacing.^24^ This observation is in line with a recent study of defects in mono- and few-layer CrSBr flakes irradiated by He ions, which also demonstrates the structural and electronic anisotropy.^25^

**Figure 1:**
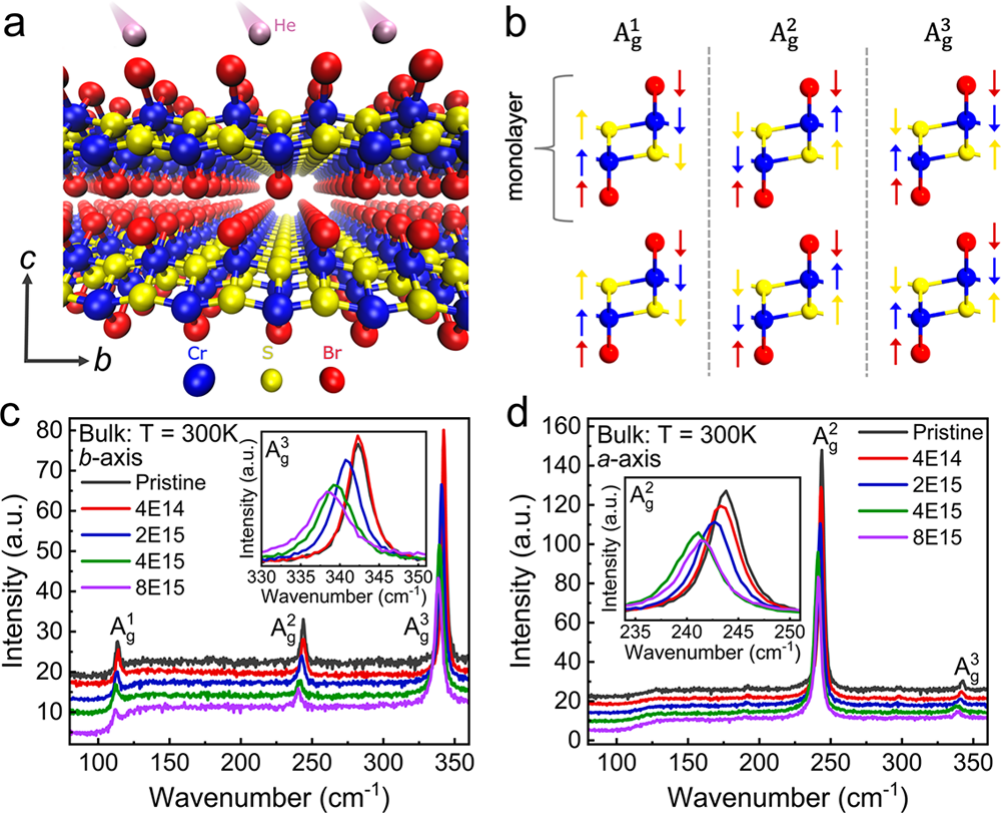
Schematic illustration of (a) He irradiation and (b) lattice vibration modes of CrSBr crystals. Color-coded arrows in (b) indicate the direction along which individual atoms move in the vibration. (c,d) Raman spectra from the 200-μm-thick bulk crystal measured after every ion irradiation step (the numbers correspond to ion fluences, e.g., 4E14 = 4 × 10^14^/cm^2^). The laser excitation was polarized along the *b*- and *a*-axis, respectively. The spectra are vertically offset for better visibility. The magnified views in the insets show a continuous reduction in wavenumber for the A_g_^3^ and A_g_^2^ modes, indicating the gradual softening of the lattice with increasing ion fluence. The measurements were taken at ambient conditions.

From SRIM (Stopping and Range of Ions in Matter) simulations,^26^ we calculate the average number of times an atom is displaced from its equilibrium lattice position during irradiation (displacement per atom, DPA), which allows us to estimate the depth profile of energy transferred from the energetic ions to the crystal matrix and thus the distribution of defects created in the crystal. The DPA of the 200-μm-thick crystal shown in Figure 2a indicates that a low concentration of defects with relatively homogeneous distribution is created in the top 4 μm of the crystal and that a high concentration of defects is expected to occur 5 to 6 μm below the surface. In the thin flake sample, on the other hand, defects are homogeneously distributed (Figure 2b). Overall, we expect a defect concentration comparable to that of the 200-μm-thick crystal created in the top 4 μm.

**Figure 2:**
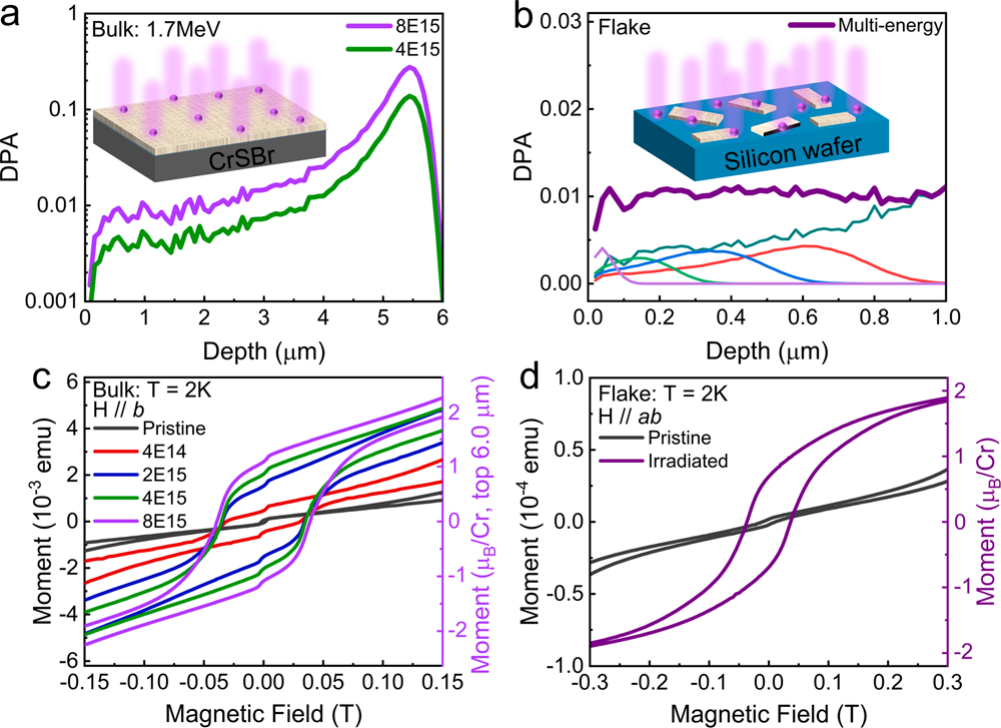
DPA for (a) the 200-μm-thick bulk sample with a fluence of 4 × 10^15^/cm^2^ (4E15) and 8 × 10^15^/cm^2^ (8E15); and (b) the flake sample (the thinner lines of different color represent the DPA induced by ions with different energy; see Table S1). The insets schematically show the sample geometry and effective thickness (the beige color). Magnetic hysteresis at 2 K for (c) the 200-μm-thick sample after irradiation at different fluences (the abrupt change near zero field is the antiferromagnetic signal from the unaffected bulk CrSBr) and (d) the flake sample after irradiation with a fluence equivalent to the maximum fluence used for the sample in (c). Measurements taken before irradiation shown for comparison are labeled pristine.

To investigate the influence of He^+^ irradiation on the magnetic properties, we measure the field- and temperature-dependent magnetization in the 200-μm-thick bulk crystal and in the thin-flake sample using SQUID-magnetometry. Figure 2c and Figure S2 show the magnetization induced in the bulk crystal by a field applied along the *b*-axis. Before irradiation, the sample exhibits the typical AFM response expected for pristine CrSBr crystals.^7^ However, after we expose the crystal to ion irradiation, magnetic hysteresis centered at zero field is observed at fields up to 0.1 T. Upon increasing the irradiation fluence, this fingerprint of FM order becomes more and more prominent. At the same time, at fields around 0.5 T, we also observe a spin-flip transition that is characteristic of AFM-ordered CrSBr (see Figure S2). This observation is fully in line with the fact that only a fraction of the volume of the 200-μm-thick crystal is altered by ion irradiation. Hence, we observe a signal that has contributions from both the irradiated and pristine regions of the sample. For a fluence of 8 × 10^15^/cm^2^, we can estimate that the saturation magnetic moment in the irradiated volume amounts to 2 μ_B_/Cr by assuming an irradiation thickness of 6 μm according to Figure 2a. After 60 days storage at ambient conditions, we remeasured our sample and found no significant change in the magnetic response (see Figure S3).

The effect of ion irradiation on the magnetic properties is much more prominent in the second sample. For the thin flakes, we observe a full transition of the magnetic ground state from the AFM to FM after ion irradiation. As demonstrated by Figure 2d and the full-field measurements in Figure S2, magnetic hysteresis centered at zero field dominates the magnetic response after irradiation and the signatures of the AFM spin-flip transition are no longer observed. The saturation magnetization approaches 2 μ_B_/Cr. Note that the saturation magnetization of Cr atoms in CrSBr is 3 μ_B_/Cr.^27,28^ For the calculation, we assumed that the saturation magnetic moment is 3 μ_B_/Cr in the pristine sample, and there is no flake loss during sample handling. The in-plane magnetic anisotropy is not observed as a sharp spin-flip transition because individual flakes are randomly oriented on this sample.

In the next step, we investigate irradiation-induced changes of magnetic properties by temperature-dependent magnetization measurements conducted with an applied magnetic field of 0.1 T. Before irradiation, both samples show a peak of magnetization at a Néel temperature of around 131 K, reflecting the AFM ground state of pristine CrSBr crystals. After irradiation with He^+^ ions, however, we observe a continuous increase of the magnetization of thin flakes as the temperature decreases (see Figure 3a), perfectly in line with our expectation of FM order in the irradiated samples. The critical temperature of the FM state is found to be 111 K. The magnetization of the 200-μm-thick bulk sample shows FM contributions as an increase in the absolute magnetization measured at low temperatures, since only a fraction of the volume is affected by ion irradiation. Nonetheless, the difference in the magnetic response after ion irradiation below 120 K is shown in Figure 3b.

**Figure 3:**
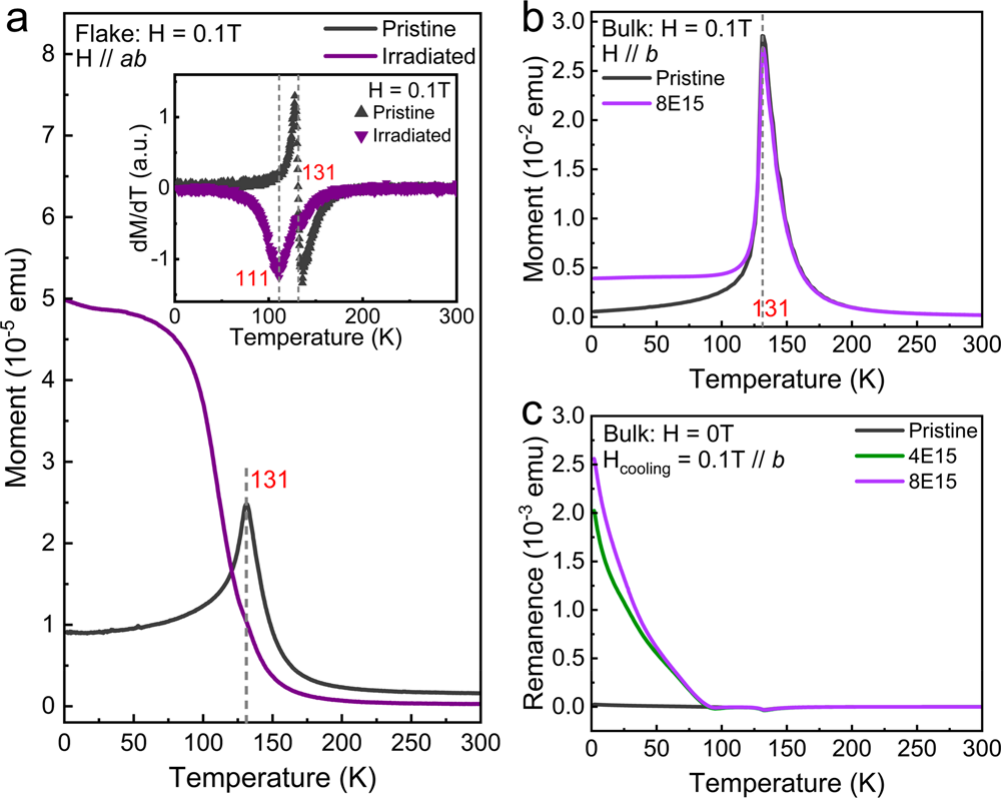
Temperature-dependent magnetization of the flake sample (a) and the bulk sample 8E15 along the *b*-axis (b) under an applied magnetic field of 0.1 T. In the inset to (a), the small kink in d*M*/d*T* at around 131 K is due to the antiferromagnetic signal of much thicker flakes, which are not fully irradiated through their thickness. Measurements on the pristine crystals are also shown for comparison. (c) Magnetic remanence of the 200-μm-thick bulk sample for two different fluences.

To characterize the remanence of the irradiation-induced FM state, CrSBr samples are cooled from room temperature, while a field of 0.1 T is applied to saturate the FM magnetization. When the temperature reaches 2 K, the magnetic field is set to zero. We then measure the remanence while increasing the temperature. The irradiated 200-μm-thick crystal exhibits sizable spontaneous magnetization below the critical temperature, as can be clearly seen in Figure 3c. We note that, while the magnetization of our bulk sample increases with irradiation fluence, the critical temperature always remains around 110 K for all fluences (see also Figure S4). Klein et al. suggested a potential defect-related phase after annealing with an onset arising also at around 110 K.^29^

As outlined above, pristine CrSBr shows a magnetic easy axis along the crystallographic *b*-axis, while the *a*- and *c*-axis correspond to intermediate and hard magnetic axes. In another ion-irradiated bulk crystal sample, we measure the temperature-dependent remanence for fields applied along the *a-*, *b-*, and *c-*axis during cool-down. As Figure S5 shows, the amplitude of the remanence is largest when the field is applied along the *b*-axis. Ion irradiation has changed the magnetic ground state, but the magnetic easy axis is preserved. This result is in good agreement with the Raman measurements presented above, indicating that the crystalline anisotropy of CrSBr remains present even after irradiation, with the largest ion fluences used in our experiments.

The transfer of energy from high-energy ions to atoms in the crystal lattice leads to the formation of defects with very high concentrations. In our case here, the SRIM simulation results in a defect concentration of around 1% for the flake sample and the top 4 μm of the bulk sample (Figure 2a,b). The most common type of defects is assumed to be Frenkel pairs—a vacancy and an interstitial atom—which are created when an atom is displaced from its equilibrium position in the lattice into an interstitial position, leaving behind an empty lattice site. This kind of lattice disorder has been shown to modify conventional 3D magnetic materials.^30,31^

We calculated the energetics of different types of vacancies and interstitials using 4 × 4 CrSBr bilayer supercells corresponding to a defect concentration of ∼0.5%. The interstitial atoms were initially placed at different positions, and the most energetically favorable structure was identified. The formation energies for Cr, S, and Br vacancies and interstitials are summarized in Table 1. Assuming that vacancies and interstitials are not interacting with each other, the Frenkel pair formation energy can be found by adding the formation energies of the isolated defects. Although the Frenkel pair formation energies are similar for three different elements, the lower values were found for S and Br, suggesting a higher concentration of these types of defects in the material. Br vacancies in CrSBr have been identified via scanning tunneling microscopy in a recent work,^29^ which is consistent with our lowest vacancy formation energy found for Br (3.59 eV/atom). Table S2 lists the total magnetic moments for various vacancies and interstitials. While the net magnetic moment in pristine CrSBr is zero, our calculations show total magnetic moments of 2.0, 6.04, and 1.05 μ_B_/supercell for S, Cr, and Br vacancies, respectively. In contrast, the S interstitial does not significantly change the magnetic moments of the pristine bilayer. The presence of magnetic character can be related to the shift between the spin-up- and spin-down-associated states in the electronic structure of the defective materials, as presented in Figures S6 and S7.

**Table 1:** Results of a Calculation of the Formation Energies of Different Vacancies (*E*_V_), Interstitials (*E*_I_), and Frenkel (*E*_F_) Defects in the CrSBr Bilayer Structures

	*E*_V_ (eV)	*E*_I_ (eV)	*E*_F_ (eV)
S	5.89	–2.18	3.71
Cr	7.76	–2.81	4.95
Br	3.59	0.45	4.03

We further investigated the preferred magnetic ordering, including the energy difference between the lowest energy AFM and FM ordering (see Figure 4). For all types of interstitials, the moments of the interstitial atoms are aligned with the Cr atoms at the bottom and top, resulting in the energetically favorable FM order. Although the energy difference depends on the exchange parameter values, the FM preference was still found for various exchange values (Table S3). The effect is more pronounced in the CrSBr bilayer with Cr interstitials. The optimized structure of the CrSBr bilayer with Cr interstitial (Figure S8) indicates that the interstitial atom chooses a position in the void between the van der Waals gap atoms forming a covalent bond between the layers, which is consistent with the previous results.^32^ The first-nearest-neighbor exchange interactions between Cr–Br–Cr and Cr–S–Cr, as well as the second-nearest-neighbor exchange between Cr–S–Cr interactions, are the main causes of the ferromagnetism of the CrSBr monolayer.^33^ The former exchange interaction is mediated by the orbital hybridization between Cr interstitials and inner Br atoms (Figure S9). As a result, the AFM coupling strength between the layers becomes weaker because of the appearance of defects.

**Figure 4:**
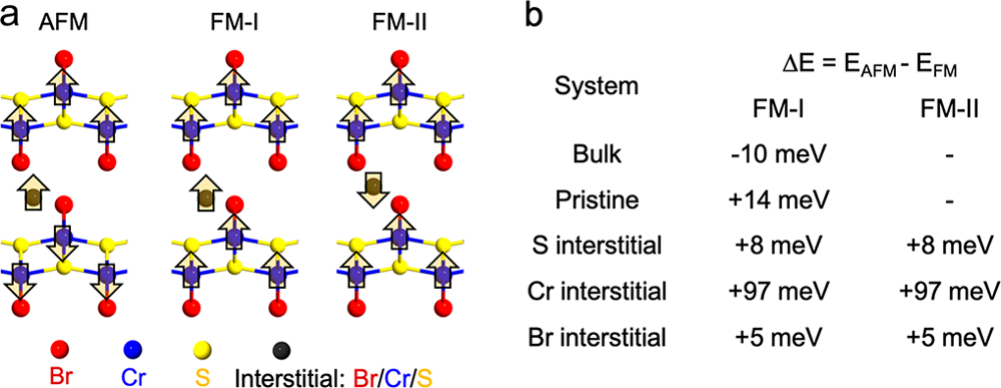
(a) Schematic representation of the magnetic configurations of interstitials in the CrSBr crystal. Regular Cr, S, and Br atoms are represented by smaller blue, yellow, and red circles, respectively. Orientations of the intralayer Cr spins are depicted by the arrows. Three spin configurations are considered for the bilayer CrSBr with interstitials. In the FM case, the magnetic moments of the interstitials can be aligned (FM-I) or antialigned (FM-II) with respect to those in the bottom and top layer. (b) The energy difference Δ*E* = *E*_AFM_ – *E*_FM_ between the AFM and FM configuration for the bulk system and bilayer with/without interstitials is given for the supercell. Interstitial density corresponds to ∼0.5%. A positive Δ*E* indicates that FM is favored over the AFM configuration.

We note that the change of the magnetic ground state observed after ion irradiation could in principle also be caused by lattice expansion, e.g., due to intercalation.^9^ However, X-ray diffraction and cross-sectional high-resolution scanning transmission electron microscopy measurements of our irradiated crystals (see Figures S10, S11) do not indicate any expansion of the lattice along the *c*-axis within the detection limit. Recent works have demonstrated the possibility of strain, hydrostatic pressure or structural phase transition in modifying the magnetic properties of CrSBr.^19,22,32^

We observe a change of the magnetic ground state of the vdW magnet CrSBr after irradiation by non-magnetic ions. The fluence-dependent remanent magnetization of the irradiation-induced FM state exists up to a critical temperature of 110 K and adapts the magnetic easy axis anisotropy of the AFM order before irradiation. The saturation magnetic moment of FM magnetization is estimated to be close to the maximum magnetic moment of the constituent Cr atoms. First-principles calculations suggest that the displacement of atoms into interstitial positions by high-energy ions is responsible for the experimentally observed transition of the magnetic ground state as the interstitials facilitate ferromagnetic coupling between the layers (structural units) of CrSBr. We note that ion implantation is a mature technology for chip fabrication. Applying proper lithography, one can create artificial lateral structures. By tuning the energy of the ions and hence the penetration depth, it may further be possible, in a vdW heterostructure, to maximize the impact of ions for CrSBr but not the material on top. As a result, our approach may be applied to modify the magnetic properties of CrSBr even after fabrication of a vdW heterostructure.

## Supporting Information Available

Methods, He irradiation parameters for the flake sample, Calculated magnetic moments for various vacancies and interstitials, The energy difference between the AFM and FM configurations, Evolution of the Raman modes of A_g_^1^ and A_g_^2^ as a function of irradiation fluence, Magnetization curves at 2 K with a large field range, Magnetization curves at 2K for irradiated flake sample and after being exposed to the ambient condition for 60 days, Magnetic remanence of the bulk sample with different irradiation fluences, Magnetic remanence of the bulk sample 8E15 along different crystallographic directions, Projected density of states (PDOS) of pristine and defective CrSBr monolayers with S, Cr, and Br vacancies, Projected density of states (PDOS) of pristine and defective CrSBr monolayers with S, Cr, and Br interstitials, Optimized structures of CrSBr bilayer with different interstitials, Projected density of states for CrSBr bilayer with Cr interstitial in FM and AFM ordering, X-ray diffraction of pristine CrSBr and sample 8E15, Cross-sectional high-resolution HAADF-STEM images and corresponding fast Fourier transforms (PDF)
