# Supplementary material for: Ferromagnetic Interlayer Coupling in CrSBr Crystals Irradiated by Ions
Source: Nano Lett. 2023 Sep 5;23(18):8468–73. doi: 10.1021/acs.nanolett.3c01920 (PMC10540254; doi:10.1021/acs.nanolett.3c01920)
Supplement: Supplementary file 1 — nl3c01920_si_001.pdf [file nl3c01920_si_001.pdf]

Ferromagnetic interlayer coupling in CrSBr crystals irradiated by ions

Fangchao Long[1,2], Mahdi Ghorbani-Asl[1], Kseniia Mosina[3], Yi Li[1,2], Kaiman Lin[1,4], Fabian Ganss[1], René Hübner[1], Zdenek Sofer[3], Florian Dirnberger[5*], Akashdeep Kamra[6], Arkady V. Krasheninnikov[1], Slawomir Prucnal[1], Manfred Helm[1,2], Shengqiang Zhou[1*]

[1]Helmholtz-Zentrum Dresden-Rossendorf, Institute of Ion Beam Physics and Materials Research, Bautzner Landstrasse 400, 01328 Dresden, Germany

[2]Technische Universität Dresden, 01069 Dresden, Germany

[3]Department of Inorganic Chemistry, University of Chemistry and Technology Prague, Technická 5, 166 28 Prague 6, Czech Republic

[4]University of Michigan-Shanghai Jiao Tong University Joint Institute, Shanghai Jiao Tong University, Shanghai, China

[5]Institute of Applied Physics and Würzburg-Dresden Cluster of Excellence ct.qmat, Technische Universität Dresden, Germany

[6]Condensed Matter Physics Center (IFIMAC) and Departamento de Física Teórica de la Materia Condensada, Universidad Autónoma de Madrid, Madrid, Spain

- Corresponding authors: florian.dirnberger@tu-dresden.de, s.zhou@hzdr.de

Supporting Information

Table of Contents

Methods

Table S1. He irradiation parameters for the flake sample

Table S2. Calculated magnetic moments for various vacancies and interstitials

Table S3. The energy difference between the AFM and FM configurations

Figure S1. The evolution of Raman-modes A_g^1 and A_g^2 as a function of irradiation fluence

Figure S2. Magnetization curves at 2 K with a large field range

Figure S3. Magnetization curves at 2 K for the irradiated flake sample and after being exposed to the ambient condition for 60 days

Figure S4. Magnetic remanence of the bulk sample with different irradiation fluences

Figure S5. Magnetic remanence of the bulk sample 8E15 along different crystallographic directions

Figure S6. Projected density of states (PDOS) of pristine and defective CrSBr monolayers with S Cr, and Br vacancies

Figure S7. Projected density of states (PDOS) of pristine and defective CrSBr monolayers with S Cr, and Br interstitials

Figure S8. Optimized structures of CrSBr bilayer with different interstitials

Figure S9. Projected density of states for CrSBr bilayer with Cr interstitial in FM and AFM ordering

Figure S10. X-ray diffraction of pristine CrSBr and sample 8E15

Figure S11. Cross-sectional high-resolution HAADF-STEM images and corresponding fast Fourier transforms

Methods

Crystal growth

CrSBr crystals were prepared by direct reaction from the elements. Chromium (99.99%, -60 mesh, Chemsavers, USA), bromine (99.9999%, Sigma-Aldrich, Czech Republic), and sulfur (99.9999%, Stanford Materials, USA) were mixed in stochiometric ratio in a quartz ampoule (35 x 220 mm) corresponding to 15 g of CrSBr. Bromine excess of 0.5 g was used to enhance vapor transport. The material was pre-reacted in an ampoule using a crucible furnace at 700 °C for 12 hours, while the second end of the ampoule was kept below 250 °C. The heating procedure was repeated two times until the liquid bromine disappeared. Afterwards, the ampoule was placed in a horizontal two-zone furnace for crystal growth. First, the growth zone was heated to 900 °C, while the source zone was heated to 700 °C for 25 hours. For the growth, the thermal gradient was reversed and the source zone was heated from 900 °C to 940 °C and the growth zone from 850 °C to 800°C over a period of 7 days. The crystals with dimensions of up to 5 x 20 mm^2 were removed from the ampoule in an Ar glovebox.

Flake exfoliation procedure

First, we picked up a CrSBr crystal and put it on the scotch tape. Then, we used plastic tweezers to crumble the CrSBr crystal to flakes and distributed them

evenly within an area of 4 mm × 4 mm. Finally, we put a clean, naturally oxidized Si wafer on the scotch tape containing the CrSBr flakes and pressed both together. After a few minutes, we removed the Si wafer from the tape and got the prepared sample.

He irradiation procedure

The CrSBr bulk sample was pasted on a piece of Si wafer. For ion irradiation, a He beam of 1.7 MeV energy with a beam size of 2 mm in diameter produced by a van de Graff accelerator was used. For the flake samples, the He ion beam with different energies (see Table S1) was scanned across the sample surface.

For the bulk sample, we estimated the irradiation volume according to the SRIM simulation result. The SRIM calculation was done by using the option "Quick Calculation of Damage". A default displacement energy of 25 eV was assumed for all elements, since, according to our knowledge, there is no literature value available for CrSBr. Our simulation is only to give a rough picture of the upper limit of damages and their depth profile. The effective depth here is about 6 μm, and the surface area of CrSBr crystal is about 1 mm × 2 mm. However, for the flake sample we cannot measure the mass and area since it contains numerous different flakes. We assumed that the saturation magnetic moment is 3 μ_B/Cr in the pristine sample to calculate the sample mass. Then, we further assumed no flake loss during sample handling and calculated the saturation magnetization after irradiation from the mass we obtained from the pristine flake sample.

Computational Methods

The energetics and magnetic properties of all point defects were investigated using spin-polarized density functional theory (DFT) as implemented in the VASP code.[1, 2] All the calculations were carried out using the Perdew-Burke-Ernzerhof exchange-correlation functional.[3] The structural models were fully optimized with an energy cut-off of 500 eV and a force tolerance of 0.01 eV Å $^{-1}$. van der Waals (vdW) interactions were taken into account using the Grimme method (DFT-D3)".[4] The point defects were modeled using a 4 × 4 × 1 supercell with a vacuum space of 20 Å in the confinement direction. The Brillouin zone of the primitive cells was sampled using 8 × 8 × 1 k-points. The electronic structure calculations were performed using the DFT + U method with an effective Hubbard value (U) of 3.0 eV for Cr atoms.

The energetics of vacancies and interstitials were assessed as $E_f = E_{defective} - (E_{pristine} \pm n_x\mu_x)$, where $E_{defective}$ and $E_{pristine}$ are the energies of the defective

and pristine supercell, respectively. n_x is the number of vacancy or interstitial atoms, and μ_x represents the chemical potential of the X species, which is considered to be the energy of the isolated atom.

Characterization Methods

Structural characterization of the as-synthesized CrSBr was performed by micro-Raman spectroscopy using a linearly polarized continuous 532 nm Nd:YAG laser for excitation. The magnetization data was collected by a superconducting quantum interference device (Quantum Design, SQUID-VSM) magnetometer.

We have carried out additional structural characterization for selected samples by X-ray diffraction of Cu $K_{\alpha 1}$ radiation on a Malvern Panalytical Empyrean and cross-sectional high-resolution scanning transmission electron microscopy (STEM) imaging with a high-angle annular dark-field (HAADF) detector employing a Talos F200X microscope (Thermo Fisher) operated at an accelerating voltage of 200 kV.

Table S1. The parameters of He irradiation for the flake sample: to achieve a homogenous *dpa* (displacement per atom) in the top around 1 μm, we applied multiple energies with different fluences. The resulted *dpa* is similar as for the bulk sample 8E15.

	Energy (keV)	*Fluence (cm^{-2})*
1	390	1×10^{15}/cm^2
2	100	1×10^{14}/cm^2
3	50	8×10^{13}/cm^2
4	20	5×10^{13}/cm^2
5	5	5×10^{13}/cm^2

Table S2. Calculated magnetic moments for 3 different vacancies and interstitials in CrSBr bilayer AFM structures. The total magnetic moments are given per vacancy/interstitial for the supercell.

	S		*Cr*		*Br*	
	Vacancy	Interstitial	Vacancy	Interstitial	Vacancy	Interstitial
Magnetic moment (μ_B)	2.0	0.0	6.04	3.17	1.05	0.99

Table S3. Energy difference $\Delta E = E_{\mathrm{AFM}} - E_{\mathrm{FM}}$ between the AFM and FM configuration is given for different on-site exchange (J) parameters of DFT+U calculations. The interstitial density is 0.5%. Here a positive sign indicates an energetically more favorable ferromagnetic ordering.

Type of system	*Hubbard parameters*	$\Delta E = E_{AFM} - E_{FM}$
Bulk (pristine)	J = 0, U = 3	-10 meV
	J = 1, U = 3	2 meV
	J = 2, U = 3	2 meV
Bilayer (pristine)	J = 0, U = 3	14 meV
	J = 1, U = 3	10 meV
	J = 2, U = 3	7 meV
Bilayer with Cr-interstitial	J = 0, U = 3	97 meV
	J = 1, U = 3	97 meV
	J = 2, U = 3	47 meV

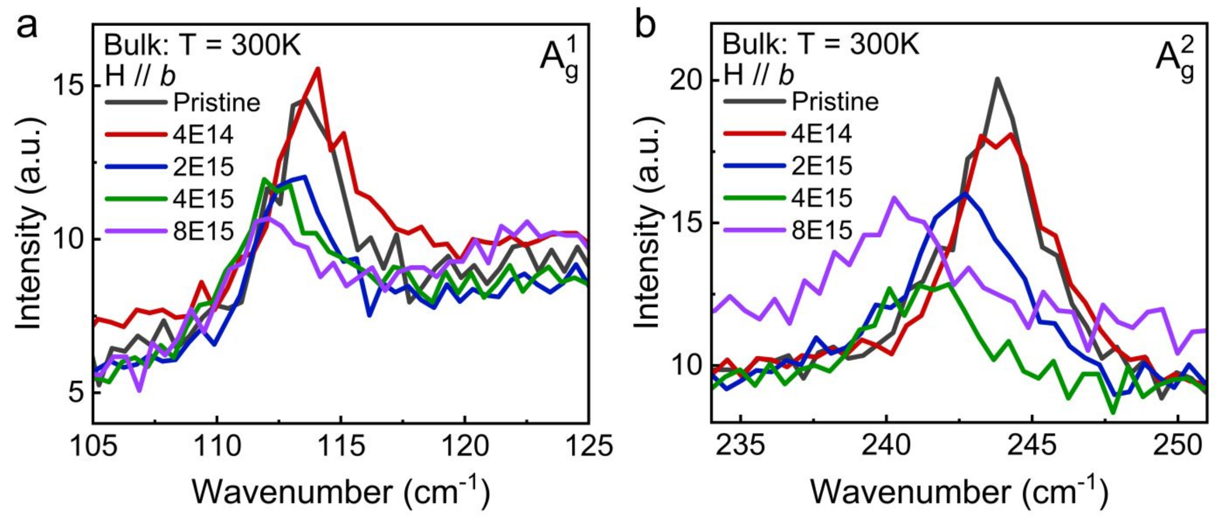

Figure S1. Evolution of the Raman modes A_g^1 and A_g^2 as a function of irradiation fluence.

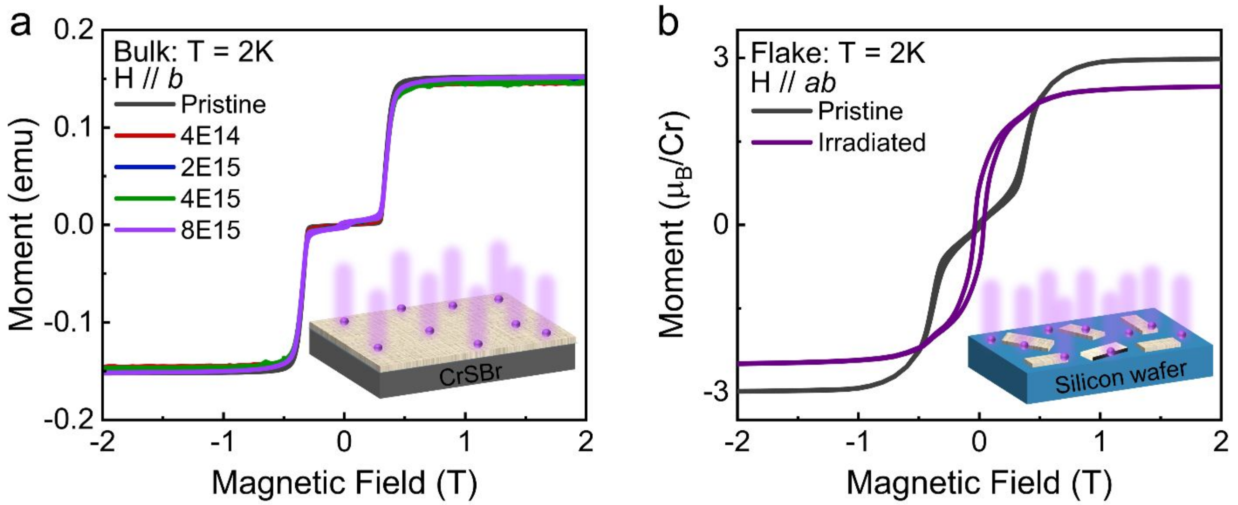

Figure S2. Magnetization curves (MH) at 2 K with a large field range for (a) the bulk sample before and after irradiation at different fluences; and (b) the flake sample before and after irradiation at an equivalent fluence of 8 $\times 10^{15}/cm^2$.

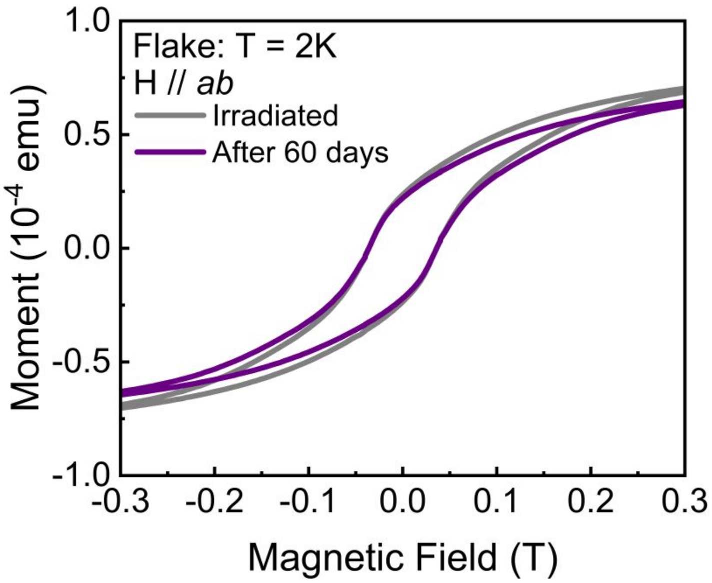

Figure S3. Magnetization curves (MH) at 2 K for the irradiated flake sample and after being exposed to the ambient condition for 60 days. There is no significant decay.

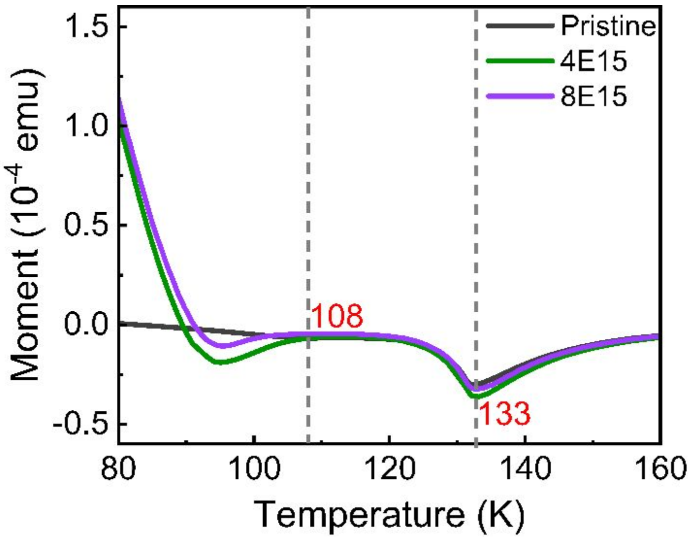

Figure S4. Magnetic remanence (the zoom-in near the critical temperature) of the bulk sample with different irradiation fluences along the *b*-axis. For the measurements, the sample was cooled down from room temperature to 2 K under the magnetic field 0.1 T along the *b*-axis, then the field was set to zero, and the magnetic remanence was measured during warming up. In the temperature range 90-110 K, one can see a weak dip, which is due to the possible negative residual magnetic field in the superconducting magnet (when being decreased from +1000 Oe to "0"), probably a few Oe. Near the critical temperature, this residual field is enough to flip some ferromagnetic nano-regions. Due to the same origin, the pronounced dip at 133 K corresponds to the Néel temperature of the non-irradiated bulk crystal. For both, bulk and flake samples, we estimated the critical temperature of the induced ferromagnetism to around 110 K.

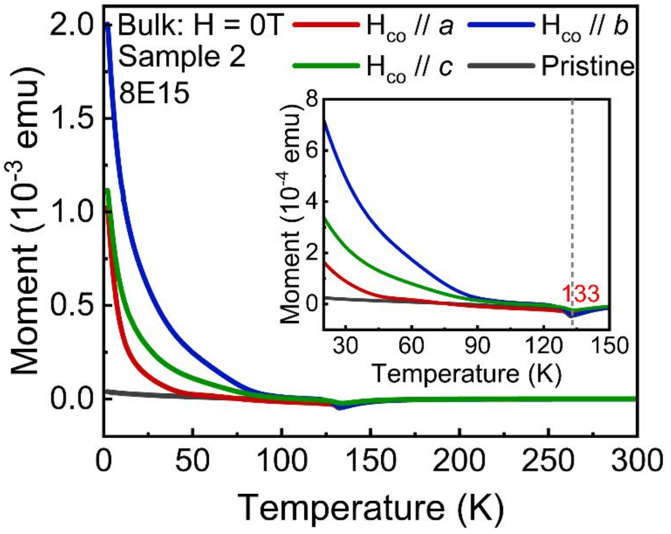

Figure S5. Magnetic remanence of the bulk sample 8E15 (a second sample) along different crystallographic directions. For the measurements, the sample was cooled down from room temperature to 2 K under the magnetic field H_{co}=0.1 T, then the field was set to zero, and the magnetic remanence was measured during warming up.

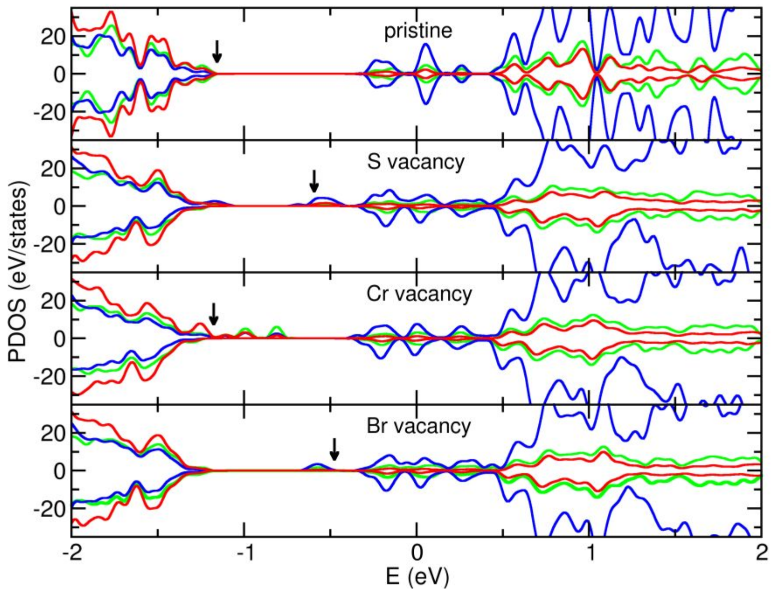

Figure S6. Projected density of states (PDOS) of pristine and defective CrSBr monolayers with S Cr, and Br vacancies. The blue, green, and red colors correspond to the projected states from the Cr, S, and Br atoms, respectively. The black arrow indicates the position of the highest occupied state.

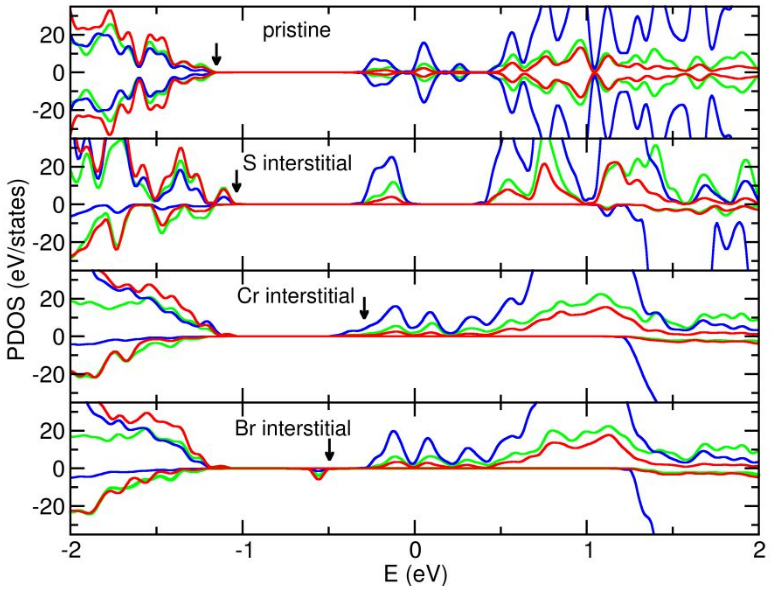

Figure S7. Projected density of states (PDOS) of pristine and defective CrSBr monolayers with S Cr, and Br interstitials. The blue, green, and red colors correspond to the projected states from the Cr, S and Br atoms, respectively. The black arrow indicates the position of the highest occupied state.

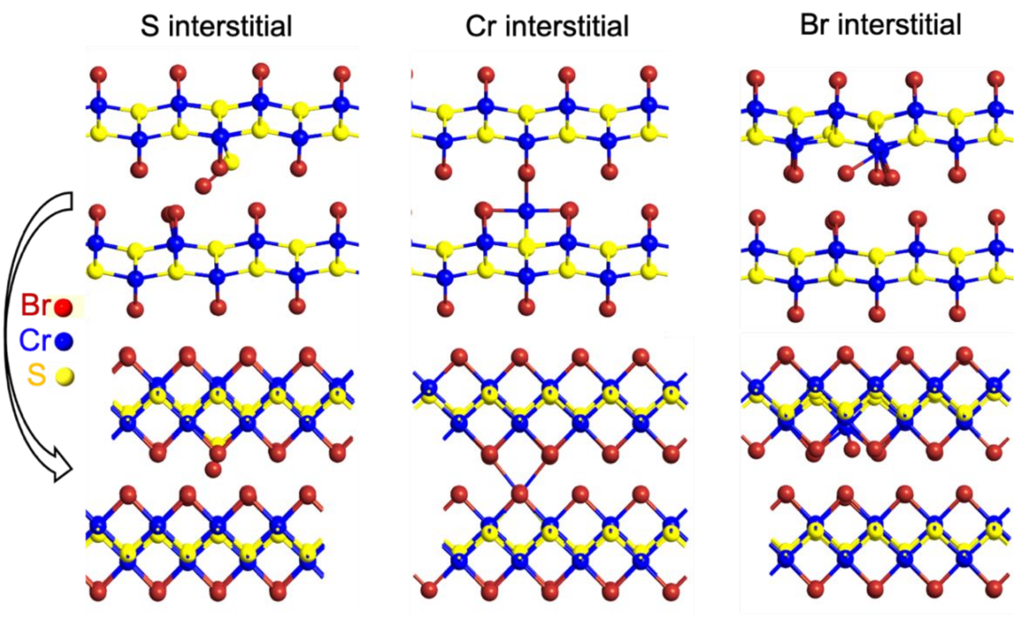

Figure S8. Optimized structures of CrSBr bilayer with different interstitials. Cr, S, and Br atoms are represented by blue, yellow, and red circles.

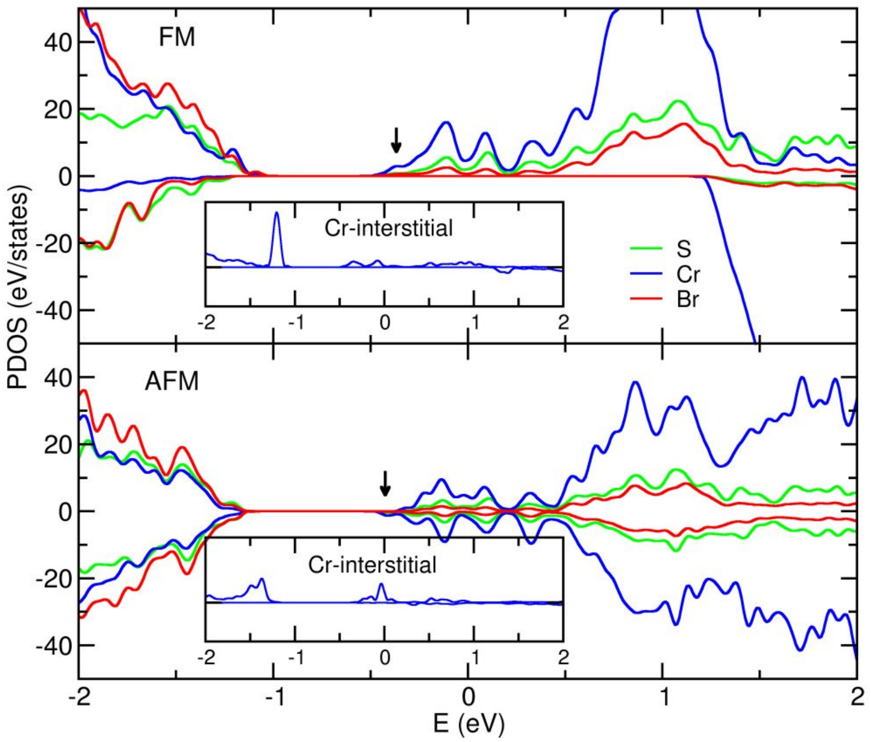

Figure S9. Projected density of states for CrSBr bilayer with Cr interstitial in FM and AFM ordering.

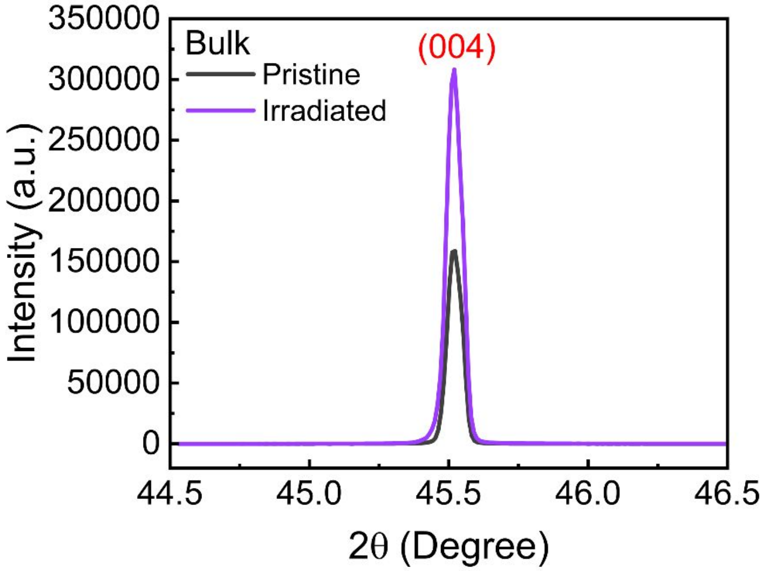

Figure S10. X-ray diffraction of pristine CrSBr and sample 8E15. By XRD, we cannot confirm any lattice expansion along the c-axis (the out-of-plane direction). It is much different in comparison with conventional semiconductor materials.[5] Here, only the CrSBr 004 reflection is shown since it is the strongest diffraction peak[6].

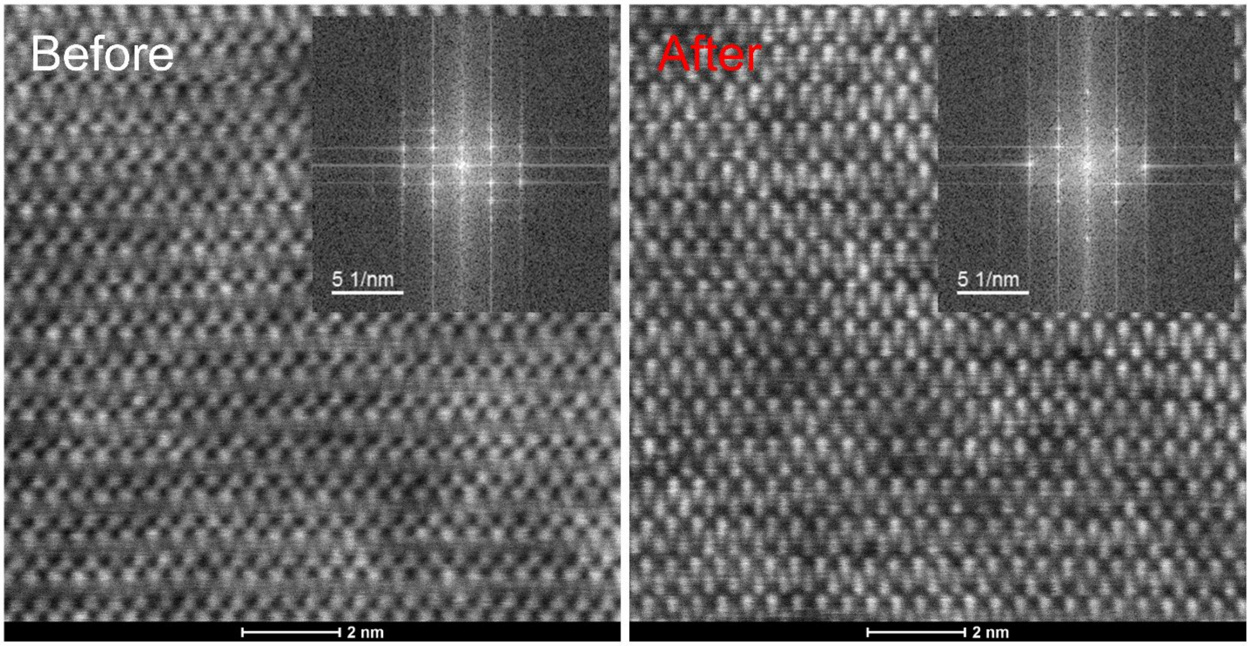

Figure S11. Cross-sectional high-resolution HAADF-STEM images and corresponding fast Fourier transforms (insets) of a new sample before (left) and after irradiation (right) with 4 MeV He ions at a fluence of $5\times10^{15}/cm^2$ which generates a similar concentration of defects (dpa) as for sample 4E15 ($4\times10^{15}/cm^2$) discussed in the main text. After irradiation, there are no structural differences visible compared to pristine CrSBr.

Supporting references

(1) Kresse, G.; Furthmueller, J. Efficient iterative schemes for ab initio total-energy calculations using a plane-wave basis set. *Phys. Rev. B* **1996,** 54, 11169–11186.
(2) Kresse, G.; Furthmueller, J. Efficiency of Ab-Initio Total Energy Calculations for Metals and Semiconductors Using a Plane-Wave Basis Set. *Comput. Mater. Sci.* **1996,** 6 (27), 15–50.
(3) Perdew, J. P.; Burke, K.; Ernzerhof, M. Generalized Gradient Approximation Made Simple. *Phys. Rev. Lett.* **1996,** 77, 3865–3868.
(4) Grimme, S.; Antony, J.; Ehrlich, S.; Krieg, H. A consistent and accurate ab initio parametrization of density functional dispersion correction (DFT-D) for the 94 elements H-Pu. *J. Chem. Phys.* **2010,** 132 (15), 154104.
(5) Holland, O. W.; Budai, J. D.; White, C. W. Uniaxial lattice expansion of self ion implanted Si. *Appl. Phys. Lett.* **1990,** 57 (3), 243-245.
(6) Liu, W.; Guo, X.; Schwartz, J.; Xie, H.; Dhale, N. U.; Sung, S. H.; Kondusamy, A. L. N.; Wang, X.; Zhao, H.; Berman, D.; Hovden, R.; Zhao, L.; Lv, B. A three-stage magnetic phase transition revealed in ultrahigh-quality van der Waals magnet CrSBr. *ACS Nano* **2022,** 16, 15917-15926.
